# Characteristics of soybean (*Glycine max*) protein hydrolysate by bromelain and its effect on inflammation and kidney disorders in gentamicin-induced male Wistar rats

**DOI:** 10.22038/ijbms.2024.78131.16885

**Published:** 2025

**Authors:** Meilinah Hidayat, Amirah Barnas, Virginia Nussy, Timothy Wantania, Sijani Prahastuti, Sriwidodo Sriwidodo, Khomaini Hasan

**Affiliations:** 1 Faculty of Medicine, Universitas Kristen Maranatha Jalan Prof.Drg. Suria Sumantri 65 Bandung 40163, Indonesia; 2 Faculty of Pharmacy, Universitas Padjadjaran Jl. Ir. Soekarno, KM 21 Jatinangor – Sumedang, 45363, Indonesia; 3 Faculty of Medicine, Universitas Jenderal Achmad Yani, Jalan Terusan Jenderal Sudirman, Cimahi 40531 PO Box 148, Indonesia

**Keywords:** Creatinine, Glycine max, hs-CRP, Phytoestrogen, Protein hydrolysates, RGD motif, Soybean

## Abstract

**Objective(s):**

Soybeans have various positive effects on health, including anti-inflammatory and preventing kidney damage. There is concern regarding the phytoestrogen content due to the high isoflavone content in soybeans. Various forms of soybean processing have been tried; in this study, the hydrolysis method will be used to obtain the active substance Arginine-Glycine-Aspartate (RGD) tripeptide in soybean protein hydrolyzed by bromelain (SPHB). The research aimed to determine the characteristics and influence of SPHB on kidney function, inflammation, body weight, and estrogen in male Wistar rats induced by gentamicin.

**Materials and Methods:**

Soybeans (*Glycine max*) are hydrolyzed using the proteolytic enzyme Bromelain from pineapples. The proteomics was investigated using Liquid-Chromatography Mass Spectroscopy Tandem (LC-MS/MS). SPHB in three doses would be tested for 28 days on male Wistar rats induced by gentamicin. The parameters measured were body weight, high-sensitive cell reactive protein (hs-CRP) levels, urea, creatinine, and estrogen levels.

**Results:**

SPHB has a low molecular weight (LMW) of 10 kDa and contains RGD in the lunasin sequence. SPHB showed no effect on body weight (*P>*0.05). The impact of SPHB on hs-CRP, urea, and creatinine showed differences significantly from the positive control, especially SPHB at a dose of 112 mg/day (*P<*0.01). Meanwhile, SPHB has almost no effect on estrogen levels.

**Conclusion:**

The administration of SPHB with LMW and contains lunasin showed decreased inflammation and kidney function parameters but did not affect body weight and estrogen levels in induced gentamicin Wistar rats.

## Introduction

So far, extensive research has been conducted on the health benefits of soybeans. These benefits include the impact of isoflavones on the risk of breast cancer (1). Soybean research results indicate benefits for several degenerative diseases such as hypercholesterolemia, heart disease, osteopenia, anti-inflammatory effects, and prevention of kidney damage (2-4). Meanwhile, many conflicting opinions regarding soybean consumption exist because of its high isoflavone content. Isoflavones are known as plant estrogens; there are concerns that long-term consumption will have a feminizing effect on men. Further research is needed to reach definitive conclusions (5).

In soybean research, various forms of soybean processing have been tried, such as soybean oil (3), fermentation (6), protein isolate or hydrolysate (4). The enzymes used in hydrolysis vary, for example, the probiotic *Lactobacillus rhamnosus* (7) or Alcalase, trans-glutaminase (8). Soy peptides containing few (4-20) amino acids have a beneficial effect against hypertension and dyslipidemia; therefore, soy peptides may benefit kidney function (9).

Soy is known as a natural ingredient that has anti-inflammatory effects. Soybeans have anti-inflammatory and antioxidant effects because they contain arginine-glycine-aspartic acid (RGD) and Bcl-2 protein (5). A polypeptide sequence containing RGD called lunasin can be isolated from soybeans. The RGD effect of lunasin is responsible for suppressing inflammation (9). Lunasin consists of amino acids, namely 43 residues, divided into four regions: 1. N-terminus (residues 1 to 22); part of unknown function; 2. a helical structure that functions to bind proteins (residues 23 to 31); 3. Arg-Gly-Asp (RGD) motif as extracellular matrix in lunasin (residues 32 to 34); 4. Carboxyl-terminus, consisting of nine aspartic acids (residues 35 to 43) involved in anti-mitotic function (11-13). In this study, we will examine the soybean protein hydrolyzed using Bromelain (SPHB) and then examine the presence of the RGD peptide. Next, the hydrolysate will be tested on rats induced with gentamicin so that their kidneys are damaged and experience inflammation. Gentamicin most frequently accumulates in proximal convoluted tubule epithelial cells (14, 15).

Our study will examine the effects of SPHB on inflammation and kidney damage. The hydrolysis method from SPHB uses a previously used method to hydrolyze green peas. Previous research has shown that green pea hydrolysate, hydrolyzed using bromelain, has a good effect on chronic kidney damage with fibrosis. The peptide sequence with anti-fibrosis effects is LERGDT, containing the RGD tripeptide group (16).

We will examine the presence of RGD lunasin in SPHB and SPHB effects on kidney function, inflammation, estrogen levels, and body weight in rats because there has been concern regarding the use of soybeans, especially their isoflavones/phytoestrogen content. Epidemiological studies show that men experience a more rapid decline in kidney function than women and are more likely to experience kidney failure (17). To determine the anti-inflammatory effect, we will examine hs-CRP. The hs-CRP is a marker of inflammation because the hs-CRP protein increases as an initial response when inflammation occurs (e.g., infection, injury, wound) (18). hs-CRP levels will increase in various inflammatory conditions that can cause infection and primary or secondary tissue damage, such as acute kidney injury (AKI) or chronic kidney disease (CKD)(19).

This research aims to know the characteristics and determine the potential of SPHB as a therapy to improve kidney function and inflammation.

## Materials and Methods

***Preparation for making soy bromelain hydrolyzed protein (SPHB)***

The preparation of protein hydrolysate is based on a modification of Patent P 00201907647 (20). A total of 500 g of soybean (*Glycine max)* powder (MDL 525 produced by CV Inti Utama Garut, Indonesia) was added to 50 g of Bromelain (*Ananas sativus*) solution (10% by weight of soybean powder) as a protease and dissolved with 2000 cc of aquadest in a beaker glass. The glass was left at 25-30 ^°^C (room temperature) with a stirrer for 12 hr. After 12 hr, the solution of SPHB was centrifuged at 4 ^°^C, 2500g. Using Whatman filter paper, the supernatant solution resulting from hydrolysis was filtered. SPHB molecular weight was analyzed using LMW Protein Ladders SDS-PAGE and standard (www.biorad.com/1610374) with a gel concentration of 12% for 120 min and a voltage of 90V (21).

Protein levels were examined using the Bradford method (22) and LC-MS/MS for proteomic analysis of SPHB (23).

***Experimental animals***

This research has received approval from the Maranatha Animal Ethics Committee (Decree No. 103/KEP/IV/2023). This test used 25 healthy male Wistar rats aged 8-10 weeks weighing 180-200 g. The rats were purchased from the School of Life Science, Bandung Institute of Technology, Indonesia. Acclimatization was carried out on all rats for seven days; each ventilated cage contained five randomly selected rats. Rats were fed standard rat chow, and water was provided *ad libitum* at room temperature maintained at 22-25 ^°^C. The light and dark cycle is attempted to alternate every 12 hr. On day 8, all rats (except the negative control group) were induced with gentamicin intraperitoneally at an 80 mg/kg BW dose for seven days (15). 

On day 15, after the gentamicin induction administration, blood was taken from all rats from the orbital vein to check all parameters as initial data. Next, SPHB treatment began to be carried out. There were five groups of rats studied, namely: negative control group (rats not given any treatment), positive control (rats only induced by gentamicin), and three treatment groups, rats that had been induced by gentamicin and given SPHB doses 1, 2 and 3 (56 mg/day, 84 mg /day, and 112 mg/day). SPHB was administered via probe orally every day for the next 28 days. The dose given was based on a modified dose from the previous study, namely a green pea protein hydrolysate dose of 100 mg/day, which improved kidney function (16). 

On day 42, all rats were terminated after being given anesthetized ketamine 100 mg/kgBW and Xylazine 10 mg/kgBW, and their blood was taken via heart puncture for parameter examination samples (24).

***Measurement of rat’s body weight***

All rats were weighed once a week. Body weight is calculated from the average body weight data at the end of week 4 (day 42) minus the average body weight before treatment (initial data, day 15).

***Measurement of urea, creatinine, hs-CRP, and estrogen levels***

Rat blood samples were taken twice before and after treatment (days 15 and 42). Blood samples are collected and placed into Eppendorf tubes. They are then centrifuged at a speed of 2500-3000 rpm for five minutes to produce serum for measuring urea levels, creatinine, hs-CRP, and estrogen. Urea, creatinine, and hs-CRP levels were examined using colorimetric principles with a Cobas Roche C311 instrument, and estrogen was examined utilizing a competition principal test with a Cobas Roche E411 instrument in a hospital laboratory in Purwakarta. Normal estrogen female rats’ levels are 11.120+1.460 pg/ml (24).

***Data analysis***

From the two data results obtained, the difference was calculated (the mean value after treatment (day 42) minus the mean value before treatment (day 15)), and SPSS version 27.0 was used for statistical analysis and classified as significant if *P*≤0.05. The Shapiro-Wilk test was performed to ensure the data were normally distributed, and the Levene test was used to measure homogeneity. Next, if the data is normally distributed, it is analyzed using ANOVA and continued with the *post-hoc* test. If the data is not normally distributed, then the Kruskal-Wallis and Mann-Whitney tests are carried out.

## Results

**Figure 1:**
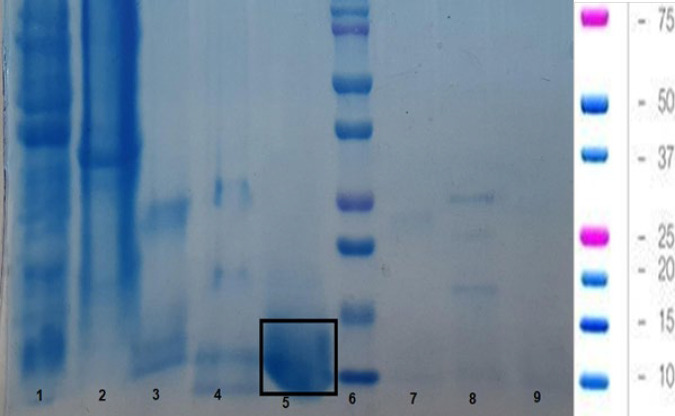
SDS-PAGE results of soybean protein hydrolysate’s molecular weight

The protein level in SPHB examined using the Bradford method was 112 mg/ml. In this study, three doses of SPHB were given to rats, namely 56 mg/day (dose 1=0.5 ml), 84 mg/day (dose 2=0.75 ml), and 112 mg/day (dose 3=1 ml). Molecular weight analysis was compared using the Low Molecular Weight (LMW) SDS-PAGE method (Figure 1). The unhydrolyzed soybean sample (line 2) was taken as a control, compared with the bromelain enzyme (line 3) and the SPHB sample (line 5). The SPHB sample (line 5) produced a band with a molecular weight of 10 kDa. In this examination, pea protein hydrolysate (lines 4 and 8) was examined compared to SPHB.

**Figure 2:**
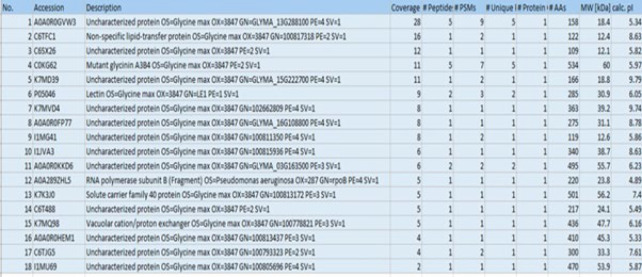
Results of proteomic analysis from soy protein hydrolysate bromelain (by search engine)

The following are the results of proteomic analysis from SPHB and amino acid analysis (Figure 2).

***Body weight***

**Figure 3:**
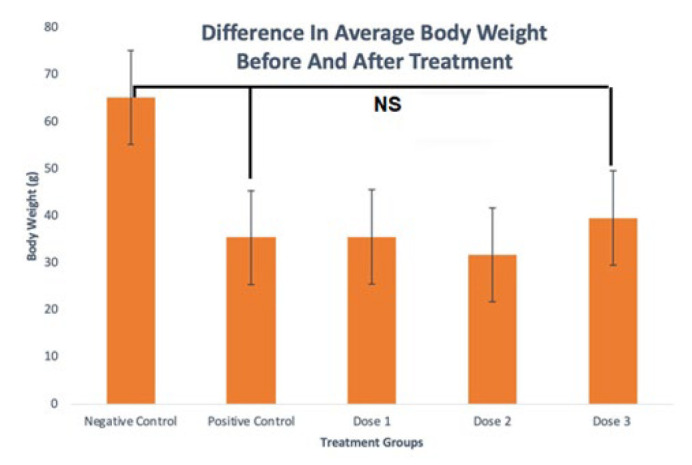
The difference in average rat’s body weight after and before SPHB treatment

The data from the weighing results were normally and homogeneously distributed, and an ANOVA analysis test was carried out. There was a relatively significant difference in mean body weight between the negative control group and the other groups. However, the calculation and analysis results of the body weight of the rats showed a non-significant difference between the positive control and the negative control or treatment groups (*P*=0.138). The highest difference in mean body weight was in the negative control group (65.25 g), followed by HPSB 112 mg (39.60 g), HPSB 56 mg (35.60), positive control (35.40 g), and HPSB 84 mg (31.80 g)(Figure 3).

*hs-CRP examination*

Using ANOVA and *post hoc* tests, analysis results showed that the positive control group differed significantly (**) (*P*=0.007) from the negative control group.

**Figure 4:**
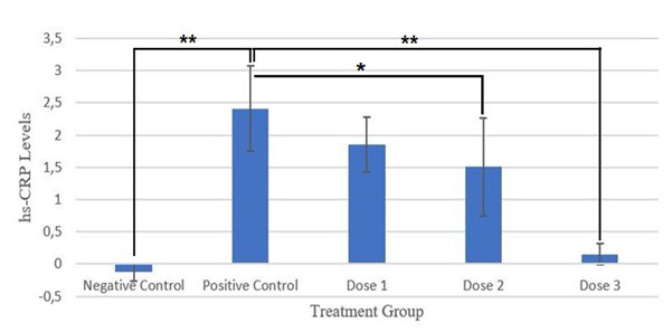
The difference in average of rats’ hs-CRP levels after and before SPHB treatment

The positive control group showed a highly significant difference (**)(*P*=0.004) from the group of dose 3 and a significant difference (*) from the group of dose 2 (*P*=0.047) (Figure 4).

***Urea examination results***

**Figure 5:**
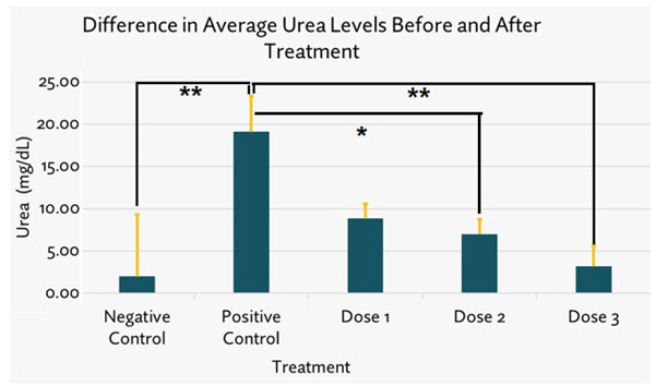
The difference in average of rats’ urea levels after and before SPHB treatment

Using Kruskal Wallis and *post hoc* Mann Whitney tests, analysis results showed that the positive control group was highly significantly different (**)(*P*=0.000) from the negative control group, indicating that gentamicin induction increased the Urea levels. A highly significant difference was shown between positive control and dose 3 (*P*=0.001)(Figure 5).

***Creatinine examination results***

**Figure 6:**
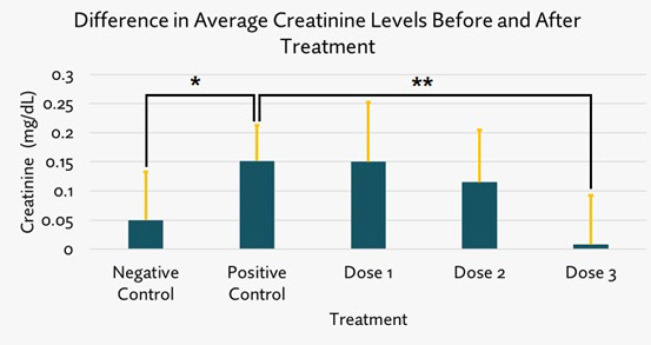
The difference in average of rats’ creatinine levels after and before SPHB treatment

Using Kruskal Wallis and Mann Whitney tests, analysis showed that the positive control group was highly significantly different (**)(*P*=0.006) from the negative control group, indicating that gentamicin induction successfully increased the creatinine levels. A highly significant difference was shown between positive control and dose 3 (*P*=0.005) and a significant difference between positive control and dose 2 (*P*=0.0265)(Figure 6).

***Estrogen level examination results***

The provision of PSHB for 28 days showed very little increase in estrogen levels. In group dose 2, the mean estrogen level was only 0.308 pg/ml, and in group dose 3 was only 0.318 pg/ml. Calculation and analysis using the ANOVA test showed a non-significant difference between the positive control and negative control or treatment groups (P>0.05)(Table 1).

## Discussion

Gentamicin is an inducer that causes inflammation by increasing Reactive Oxygen Species (ROS), which can trigger the synthesis of pro-inflammatory cytokines such as IL-1β and TNF-α, stimulating inflammatory reactions and kidney cell damage (25). One marker that can be used in inflammatory diseases such as chronic kidney disease is C-reactive protein (CRP)(26). The positive control group in this study produced a mean difference in hs-CRP levels much higher than the negative control group, indicating that gentamicin induction successfully increased the creatinine levels. The positive control also differed from the groups of doses 3 and 2. These results suggest that the doses that can be used to reduce hs-CRP levels are doses of 84 mg and 112 mg. Soybean protein hydrolysate in a dose-dependent manner has been shown to reduce inflammation by lowering the expression and mRNA level of the cytokines IL-6, TNF-α, and IL-1β (27, 28). The noteworthy study by Kim (2021) explains that the existence of the RGD motif in soybean hydrolysate contributes to the anti-inflammatory effect (6). Soybean protein hydrolysate contains the tripeptide RGD, which is the helical region of lunasin. The RGD tripeptide as an anti-inflammatory will inhibit the production of pro-inflammatory cytokines such as TNF-α and IL-6, blocking the NF-kβ factor pathway in cells induced by lipopolysaccharide (LPS) without affecting cell survival. Lunasin has also been shown to inhibit the production of PGE2, NO, and COX-2 in macrophages (29).

SPHB was proven to have an anti-inflammatory effect, as indicated by a decrease in hs-CRP levels compared to the negative control (Figure 3). This is likely due to the presence of the RGD tripeptide in SPHB, which has an anti-inflammatory effect. Indeed, we have not measured the levels of RGD contained in SPHB. Still, the impact of pure RGD, whose peptide is specially synthesized, has been proven as a comparative anti-fibrosis control in preventing kidney damage in CKD in the previous study, reducing levels of fibronectin and TGF-β 1 in research on the effects of green peas protein hydrolysate using bromelain in SV40 cells of kidney MES 13 cells induced by high glucose (16).

Kidney damage is measured using urea and creatinine parameters. The heavy metal concentrations inhibited 50% of enzyme activity (29). In a 2015 research, the induction of gentamicin at a dose of 80 mg/kg BW/day injected intraperitoneally for eight days showed clear kidney damage, which was demonstrated by an increase in blood urea and serum creatinine levels (30). On histopathological preparations, there are prominent changes in the proximal cortical segment, extensive necrosis with dilatation, vascular degeneration, epithelial desquamation, and inflammatory cells. There is increased apoptosis in tubular cells and increased oxidative stress in renal tubular cells (31). In this study, after induction using gentamicin, urea and creatinine levels in the positive control group increased significantly, indicating proof that gentamicin causes impaired kidney function. The SPHB treatment group, especially the 112 mg dose, reduced urea and creatinine levels. SPHB contains the tripeptide RGD in the sequence lunasin. Zhang *et al*. 2019’s research showed that RGD was present on the surface of Mesenchymal Stem Cell Extra Vesicles (MSC-EVs), which has an antifibrotic effect on chronic inflammation. The presence of microRNA let-7a-5p in MSC-EVs causes increased cell autophagy and decreased cell apoptosis in AKI. RGD hydrogel facilitated let-7a-5p-containing EVs from MSCs, ameliorating acute kidney damage (32). Various scientific studies show that lunasin has anti-inflammatory and antioxidant properties (33), so SPHB-containing lunasin can potentially reduce damage and inflammation to the kidneys.

In general, consumption of gentamycin can inhibit the increase in body weight in rats. Suppression of weight gain is due to the nephrotoxic effects of gentamicin. In the inflammatory process in the kidneys, pro-inflammatory mediators will be released. TNF-α, one of the mediators released during chronic inflammation, can inhibit myocyte differentiation, causing muscle wasting (34). Increased leptin in the inflammatory process causes hypercatabolism, inhibition of lipogenesis, and increased lipolysis, which causes inhibition of weight gain (35).

Our original hypothesis was that SPHB could increase the body weight of rats induced by gentamicin. However, the results of body weight after SPHB treatment showed that there were no significant differences between treatment groups (*P*>0.05), even though there were quite significant differences between the negative control group and the four groups that received gentamicin induction. This may occur because the administration of gentamicin has been shown to cause kidney damage (15, 30) but has not reduced body weight. In the treatment groups, which were not different from the positive control, it is possible that the dose of SPHB given was not high enough to increase the body weight of rats. Results of a meta-analysis study showed that the group of rats given more than 40 g/day of soy protein showed a significant increase in rats’ body weight (35).

The idea that the phytoestrogens in soybeans can feminize men is why we examined increased estrogen levels in this study. A meta-analysis analyzed clinical data from 38 clinical studies deemed suitable to measure the effects of soybean on estrogen hormone levels. This study concludes that soybeans have no effect on estrogen or testosterone levels in men. (36, 37). In this study, we wanted to see how much estrogen levels increased in rats if they were given SPHB for 28 days. The results showed that SPHB administration caused a very low increase in mean estrogen levels (*P*>0.05)(Table 1).

A previous meta-analysis study also concluded that no evidence from 9 clinical studies in men showed that phytoestrogen exposure affected circulating estrogen levels. Isoflavones have not been shown to influence sperm or semen parameters in adult men. However, the results of the study show that isoflavones can increase the risk of erectile dysfunction in animals. This does not apply to humans because there are differences in the metabolism of isoflavone between humans and rodents, depending on the length of treatment (more than three months) and the high amount of isoflavones consumed (38). From the results of a clinical review article, side effects of isoflavone estrogen were not seen on the endometrium or estrogen levels in women, nor on testosterone or estrogen levels, sperm, or semen parameters in men. This article concludes that no evidence supports isoflavones as endocrine disruptors (39).

Description (from left): 1. Pea powder, 2. Soybean powder, 3. Bromelain enzyme, 4. Pea hydrolysate solution, 5. Soybean hydrolysate solution (Molecular weight ~10 kDa), 6. Ladders, 7. Bromelain enzyme dilution 10×, 8. Pea hydrolysate solution dilution 10x, 9. 10x dilution of soybean hydrolysate solution

From the proteomic results, 18 protein accessions are listed in the figure. In the protein, A0A0R0GVW3 uncharacterized protein Glycine max (S1=Soy) OX=3847 · Gene: GLYMA_13G288100 PE=4 SV=1, the Glycine max-domain contains 158 amino acids, 18.4 kDa, 5.34 Pi. The 158 amino acids are as follows:

MTKFTILLISLLFCIAHTCSASKWQHQQDSCRKQLQGVNLTPCEKHIMEKIQGRGDDDDDDDDDNHILRTMRGRINYIRRNEGKDEDEEEEGHMQKCCTEMSELRSPKCQCKALQKIMENQSEELEEKQKKKMEKELINLATMCRFGPMIQCDLSSDD

Among the 158 amino acids, 43 amino acid residues were known as the lunasin sequence, which consisted of four sequence sections: the N-terminus section, the putative helical region, the RGD tripeptide, which was thought to be the anti-fibrosis active substance in lunasin, and finally, the Carboxyl-terminus.

SKWQHQQDSCRKQLQGVNLTPCEKHIMEKIQGRGDDDDDDDDD

SKWQHQQDSCRKQLQGVNLTPC: N-terminus

EKHIMEKIQG: putative helical region. Conserved region in chromatin binding proteins: helical region to core histones.

RGD: attachment of Lunasin to the ECM.

DDDDDDDD: 8 negatively charged Asp (D) residues at Carboxyl-terminus

Dose 1: 56 mg/d, Dose 2: 84 mg/d, Dose 3: 112 mg/d

* Significant (*P<*0.05), ** highly significant (*P<*0.01)

Dose 1: 56 mg/d, Dose 2: 84 mg/d, Dose 3: 112 mg/d

* Significant (*P<*0.05), ** highly significant (*P<*0.01)

Dose 1: 56 mg/d, Dose 2: 84 mg/d, Dose 3: 112 mg/d

* Significant (*P<*0.05), ** highly significant (*P<*0.01)

Dose 1: 56 mg/d, Dose 2: 84 mg/d, Dose 3: 112 mg/d

* Significant (*P<*0.05), ** highly significant (*P<*0.01)

**Table 1:** The difference in mean estrogen levels after and before SPHB treatment

Average Estrogen Level (pg/ml)	Differences
Negative control	ND
Positive control	ND
Dose 1	ND
Dose 2	0.308 (NS)
Dose 3	0.318 (NS)

ND: non-detectable, NS: non-significant (*P>*0.05)

Dose 1: 56 mg/d, Dose 2: 84 mg/d, Dose 3: 112 mg/d

## Conclusion

Soybean protein hydrolysate bromelain, with molecular weight <15 kDa, contains RGD in lunasin sequence and shows promising potential for improving kidney function as indicated by a reduction in kidney function parameters, reducing inflammation as indicated by a decrease in hs-CRP levels. However, the provision of SPHB at doses of 56, 84, and 112 mg for 28 days did not affect estrogen levels and body weight of male-induced gentamicin Wistar rats.

## Declaration

We have not used any AI tools or technologies to prepare this manuscipt.
